# *Hot* to Go: The impact of protein nitrosylation on plant fertility

**DOI:** 10.1093/plphys/kiaf011

**Published:** 2025-01-08

**Authors:** Anna Moseler

**Affiliations:** Assistant Features Editor, Plant Physiology, American Society of Plant Biologists; INRES-Chemical Signalling, University of Bonn, 53113 Bonn, Germany

Successful reproduction is essential for species survival and crop breeding. Fertility can be impacted at multiple levels, including gene expression, protein activity, and cellular processes, all of which are tightly regulated by post-translational modifications (PTMs) (Millar et al. 2019; Zhou et al. 2023). *S*-nitrosylation (also known as *S-*nitrosation) is a type of protein PTM in which a nitric oxide (NO) group is added to the sulfur atom of a cysteine residue. *S*-nitrosylation can alter protein function, stability, and protein interaction with other molecules. Nitrosoglutathione (GSNO) serves as a major buffer for NO, and it can be reduced by the *S*-nitrosoglutathione reductase (GSNOR). GSNOR therefore plays an important role in the regulation of levels of NO and consequently *S-*nitrosothiols (Feechan et al. 2005). Previous observations that NO plays a role in plant growth, development, and responses to environmental stress have led to the proposal that *S*-nitrosylation is important for cellular signaling. Analysis of *gsnor* mutants showed that disruption of NO homeostasis also affects plant fertility (Lee et al. 2008).

In this issue of *Plant Physiology*, Treffon and Vierling address the role of protein *S-*nitrosylation in plant fertility. They analyzed the *S-*nitrosoproteome and the total quantitative proteome of different floral tissues in *Arabidopsis thaliana* (Treffon and Vierling 2024). To define the protein targets of *S-*nitrosylation, the authors used the null mutant *hot5-2*, which encodes GSNOR and which has only 21% of GSNOR activity containing thereby increased amounts of protein *S*-nitrosothiols (R-SNOs) compared with the wild type (WT) (Feechan et al. 2005).

First, the authors established a method to detect R-SNOs in planta based on the specific reaction of organomercury with *S*-nitrosocysteine, forming a stable thiol-mercury bond. Samples in which the SNO was cleaved by UV photolysis before reaction with organomercury were used as a control. After the tryptic digestion of the trapped proteins, the peptides were eluted by mild performic acid with simultaneous oxidation of the previously *S*-nitrosylated cysteine thiols to sulfenic acids. Site-specific identification of the modified cysteines was then performed by liquid chromatography coupled to tandem mass spectrometry. Using this method, Treffon and Vierling first identified R-SNOs in whole pre-anthesis inflorescences of *hot5-2* and WT plants. The authors identified a total of 1102 *S*-nitrosylated proteins, of which 1049 *S*-nitrosylated proteins were specifically enriched in *hot5-2*. Comparison with a previously published *S-*nitrosoproteome of Arabidopsis seedlings by Hu et al. (2015), in which 928 R-SNOs were identified, revealed that 728 proteins were specific to the inflorescence dataset (Fig. A). KEGG pathway analysis of the entire dataset of 1102 *S*-nitrosylated proteins showed enrichment of proteins involved in carbon fixation, TCA cycle, oxidative phosphorylation, and protein degradation. Analysis of the 728 inflorescence-specific R-SNOs revealed a similar enrichment pattern.

**Figure.**
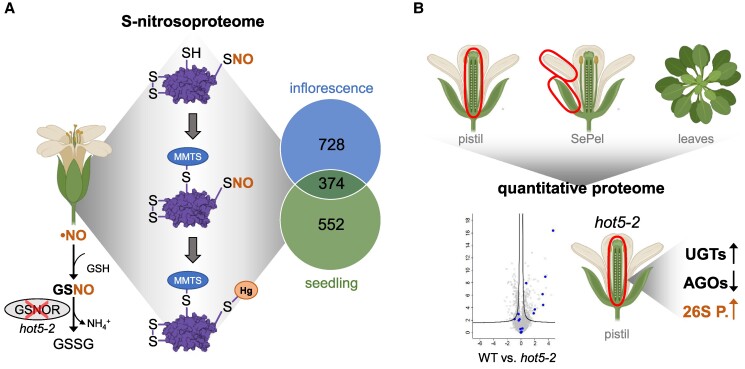
*S*-nitrosoproteome and quantitative proteome analysis of different tissues of Arabidopsis *hot5-2* and WT plants. **A)** NO reacts with GSH to form GSNO, which can either promote protein *S*-nitrosylation or be degraded to oxidized glutathione (GSSG) and ammonium (NH_4_^+^) by GSNOR. *hot5-2*, which encodes GSNOR, is a null mutant. Inflorescences were used to analyze the *S*-nitrosoproteome of *hot5-2* and WT plants. After extracting proteins from tissues, reduced thiols (-SH) were blocked with methyl methanethiosulfonate (MMTS, blue filled circles). Afterwards, *S*-nitrosylated residues (-SNO) were captured with p-aminophenylmercuric-acetate coupled to agarose beads (Hg, orange filled circles). Comparison of the *S*-nitrosoproteome with a previous published dataset from seedlings revealed 728 flower-specific *S-*nitrosylated proteins. **B)** Analysis of the *hot5-2* and WT quantitative proteome was performed from pistils and SePel as well as a previous published dataset of leaves. Comparison of the datasets from different tissues of WT and *hot5-2* and evaluation of the most highly up- or downregulated proteins in the 3 different tissues using volcano plot analysis (left diagram) showed an upregulation of UGTs and downregulation of argonaute proteins (AGOs) in *hot5-2* pistils (black arrows). An upregulation and *S*-nitrosylation was shown for proteins associated with the 26S proteasome (26S P.) in the *hot5-2* pistils (orange arrow) (modified after Treffon and Vierling 2024).

As the enriched proteins in the *hot5-2* mutant could also be caused by differences in protein abundance, the authors carried out a more detailed analysis of the entire quantitative proteome in floral tissues to investigate the relationship between protein *S*-nitrosylation and protein levels and how variations in protein levels might contribute to the fertility defects observed in *hot5-2*. Comparison of the quantitative proteome of isolated pistils, the female reproductive part of a flower, with sepals and petals (SePels), the outer sterile whorls of the flower, revealed that 6% (193 proteins; pistil) and 4% (149 proteins; SePel) were differentially regulated in *hot5-2* compared with the WT control. To further identify proteins that are exclusively enriched or depleted in a specific tissue as well as overall differences between *hot5-2* and WT, Treffon and Vierling included a previously published quantitative proteomics dataset obtained from leaves of 4- to 6-week-old Arabidopsis plants (Treffon et al. 2021) (Fig. B). In *hot5-2* pistils, one of the most upregulated protein groups belongs to the UDP-glycosyltransferase (UGT) protein family, with 7 out of 17 UGTs significantly upregulated. These enzymes use UDP sugars for glycosylation and have been shown to be involved in the regulation of plant hormone activity (Wang 2009). Two argonaute proteins, AGO5 and AGO9, were significantly downregulated in *hot5-2* pistils. AGOs are involved in the transcriptional and post-transcriptional gene silencing by binding small RNAs to form RNA-induced silencing complexes (Zhang et al. 2015). However, these proteins and the 7 UGTs were not found in the *S*-nitrosoproteome dataset.

Proteins that were both *S*-nitrosylated and upregulated in the *hot5-2* mutant included class 4 aldo-keto reductases (AKRs) and 26S proteasomal proteins. AKRs were recently identified as enzymes that may play a role in regulating NO homeostasis by degrading GSNO (Stomberski et al. 2019; Treffon et al. 2021). Treffon and Vierling found that AKR4C8 and AKR4C9 in *hot5-2* leaves were upregulated and *S*-nitrosylated, while AKR4C10 and AKRC411 were upregulated in *hot5-2* inflorescences. Regarding the 26S proteosomal proteins, which belong to the protease complex responsible for regulated protein degradation (Bard et al. 2018), the team found particularly in *hot5-2* pistils increased protein abundance of several 26S proteosomal subunits, such as the regulatory particle proteins and core proteins, but also proteins involved in proteasome activation and assembly. Ten out of 16 proteins were also found to be *S*-nitrosylated. Further analysis of proteolytic activities in tissue extracts revealed that the trypsin-like proteolytic activity in the *hot5-2* pistils was 42% higher than in the WT pistil sample. This increased proteolytic activity in *hot5-2* pistils was further confirmed by gel electrophoresis.

Taken together, this work provides a comprehensive *S*-nitrosoproteome dataset and reveals hundreds of flower-specific *S*-nitrosylated proteins, providing initial insights into the mechanisms causing the defects in female gametophyte development in *hot5-2*. The *S*-nitrosoproteome combined with the quantitative protein abundance profiling revealed thereby several differentially regulated proteins in the reproductive tissue including upregulated UGTs and downregulated AGOs. In addition, upregulation and *S*-nitrosylation of multiple 26S proteasome subunits in *hot5-2* pistils link a putative mechanism between protein quality control and NO disruption. However, the direct correlation with lower fertility of the *hot5-2* mutant has yet to be determined. It should be noted that the shotgun mass spectrometry used in the study would likely have missed proteins with low abundance. Also, it is conceivable that the increased levels of reactive nitrogen species in the *hot5-2* mutant may result in off-targets that are not physiologically relevant. However, the identification of *S*-nitrosylation targets opens up opportunities for future research into the impact of *S*-nitrosylation on protein activity and its biological role in plant growth and reproduction.

## Data Availability

No new data included in this article.
